# Diverged and Active Partitiviruses in Lichen

**DOI:** 10.3389/fmicb.2020.561344

**Published:** 2020-10-21

**Authors:** Syun-ichi Urayama, Nobutaka Doi, Fumie Kondo, Yuto Chiba, Yoshihiro Takaki, Miho Hirai, Yasutaka Minegishi, Daisuke Hagiwara, Takuro Nunoura

**Affiliations:** ^1^Research Center for Bioscience and Nanoscience (CeBN), Japan Agency for Marine-Earth Science and Technology (JAMSTEC), Yokosuka, Japan; ^2^Laboratory of Fungal Interaction and Molecular Biology (Donated by IFO), Department of Life and Environmental Sciences, University of Tsukuba, Tsukuba, Japan; ^3^Microbiology Research Center for Sustainability (MiCS), University of Tsukuba, Tsukuba, Japan; ^4^Nippon Gene Co., Ltd., Toyama, Japan; ^5^Super-cutting-edge Grand and Advanced Research (SUGAR) Program, JAMSTEC, Yokosuka, Japan

**Keywords:** RNA virus, lichen, dsRNA, partitivirus, viral metagenome

## Abstract

The lichen is a microbial consortium that mainly consists of fungi and either algae (Viridiplantae) or cyanobacteria. This structure also contains other bacteria, fungi, and viruses. However, RNA virus diversity associated with lichens is still unknown. Here, we analyzed RNA virus diversity in a lichen dominated by fungi and algae using dsRNA-seq technology and revealed that partitiviruses were dominant and active in the microbial consortium. The *Partitiviridae* sequences found in this study were classified into two genera, which have both plant- and fungi-infecting partitiviruses. This observation suggests that the lichen provides an opportunity for horizontal transfer of these partitiviruses among microbes that form the lichen consortium.

## Introduction

The lichen is a symbiotic microbial consortium that is mainly composed of a fungus (mycobiont) and photosynthetic partner (photobiont), either green algae (Viridiplantae) or cyanobacteria, or both, that harbor chlorophyll. The phototrophic partner feeds organic compounds to the fungus. In contrast, the fungus protects minute photosynthetic cells from environmental stresses, such as drought and nutrient deficiencies, and provides a suitable environment for photosynthesis and gas exchange (White and Torres, 2009). In general, these organisms form delicate structures and stratification of the lichen thallus with other microorganisms including endophytic fungi, other algae, and bacteria. For example, a typical foliose lichen thallus reveals four zones of interlaced fungal filaments (Moore et al., 2020). The uppermost zone, called the cortex, is formed by densely interwoven hyphae forming an outer protective tissue layer. The algal cells occur in a zone beneath the cortex embedded in a dense hyphal tissue. The third zone, called the medulla, is formed by loosely interwoven fungal hyphae without algal cells. The lower surface of the thallus is called the lower cortex and may consist of densely packed fungal hyphae. It is noteworthy that an endosymbiotic interaction was recently reported; green algal cells can enter fungal cells under certain conditions (Du et al., 2019). Given that pathogenic and mutualistic biotrophic interactions between plants and fungi are common on Earth (Kohler et al., 2015), the plant– and green algae–fungal interaction has a long history (Remy et al., 1994; Honegger et al., 2013; Lutzoni et al., 2018; Nelsen et al., 2020).

RNA viruses associated with lichen have been reported. Partial sequences of Cytorhabdovirus (family *Rhabdoviridae*) and Apple mosaic virus (family *Bromoviridae*) were detected by RT-PCR from lichens (Petrzik et al., 2013). In addition, *Chrysothrix chrysovirus 1* (family *Chrysoviridae*) and *Lepraria chrysovirus 1* (family *Chrysoviridae*) were identified from *Chrysothrix chlorina* and *Lepraria incana* lichens, respectively, and the former was observed in accompanying endolichenic fungus in the lichen by *in situ* hybridization (Petrzik et al., 2019). Although these studies revealed the presence of RNA viruses in lichens, the RNA viral community in lichens is still unknown. In this study, we identified viruses related to the family *Partitiviridae*. *Partitiviridae* are bisegmented dsRNA viruses that infect plants, fungi, or protozoa. Five viral genera (*Alphapartitivirus*, *Betapartitivirus*, *Gammapartitivirus*, *Deltapartitivirus*, and *Cryspovirus*) have been established in this family. Among them, host of *Gammapartitivirus* and *Deltapartitivirus* are identified as fungi and plant, respectively, and some of the species in *Alphapartitivirus* and *Betapartitivirus* infect fungi or plant (Nibert et al., 2014).

The metagenomic approach is a powerful tool to understand RNA virus diversity (Shi et al., 2016, 2018). To date, several methods to construct (meta)genomic sequencing libraries from RNA viral genomes have been established and applied to environmental samples (Culley et al., 2006; Roossinck et al., 2010; Steward et al., 2013; Urayama et al., 2016, 2018b; Decker et al., 2019). Among them, fragmented and primer ligated dsRNA sequencing (FLDS) has remarkable advantages in construction of complete viral genomes (Urayama et al., 2018a; Fukasawa et al., 2020; Kadoya et al., 2020). In this study, we applied dsRNA-seq and ssRNA-seq techniques to elucidate RNA virus diversity associated with a lichen.

## Materials and Methods

### Sample Collection

**FIGURE 1:**
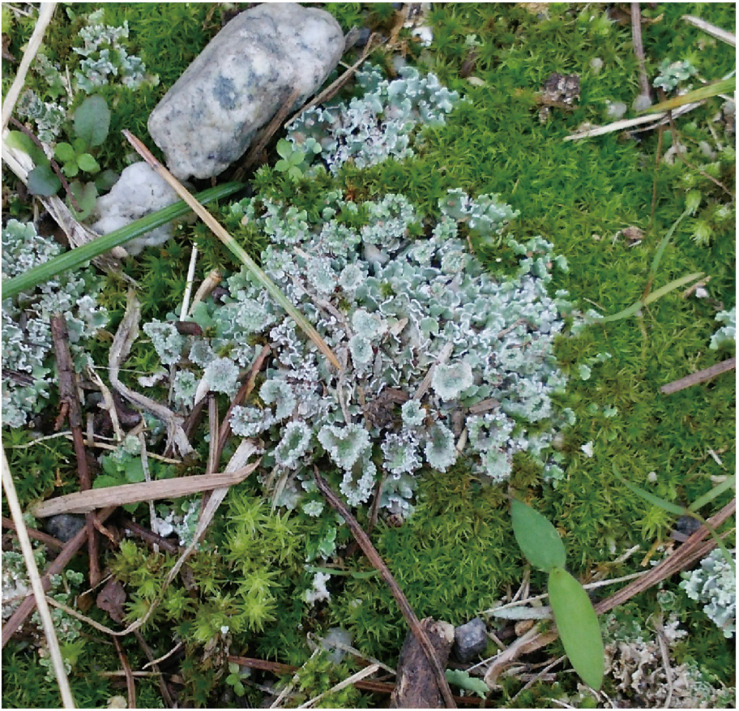
The lichen community from which samples were taken in this study.

Lichen on sand mud in Toiya-machi, Toyama (36.7007°N and 137.2475°E) was sampled in February 2019 (Figure 1). Sample was stored at −80°C until further analysis. A voucher for this lichen was not preserved due to stored sample quality.

### Extraction and Purification of dsRNA and ssRNA

The lichen sample was disrupted in liquid nitrogen in a mortar. For dsRNA extraction and purification, the ISOVIRUS (Nippon Gene, Tokyo, Japan) kit was used. In brief, total RNA was extracted in the extraction buffer, and dsRNA was purified with cellulose resin and eluted by nuclease-free water after DNase I treatment according to the manufacturer’s protocol. To obtain total RNA, the TRIzol Plus RNA Purification Kit (Invitrogen, Carlsbad, CA, United States) was used according to the manufacturer’s protocol. Total RNA was treated with DNase I (Invitrogen) and further purified using the RNA Clean and Concentrator-5 Kit (Zymo Research, Orange, CA, United States).

### cDNA Synthesis and Sequencing Library Construction

In this study, we applied FLDS to obtain RNA viral sequences. dsRNA, which is a molecular marker of RNA virus infection, is used as a template for cDNA synthesis because cellular long dsRNA is a replicative intermediate of ssRNA virus as well as genome of dsRNA virus. Simultaneously, full-length cDNA is synthesized by applying template-switching activity of the reverse transcriptase with an oligonucleotide primer against adapter-ligated dsRNA fragments, which enabled us to obtain complete genome sequences of non-retro RNA viruses.

Sequencing libraries were constructed as described previously (Urayama et al., 2016, 2018b). dsRNA was converted to cDNA using the FLDS method. In brief, DNase I and S1 nuclease-treated dsRNA was fragmented and ligated to a DNA adapter. With an oligonucleotide primer against the adapter sequence, cDNA was synthesized by using the SMARTer RACE 5′/3′ Kit (Takara Bio, Kusatsu, Japan). ssRNA was applied to the SMARTer Universal Low Input RNA Kit (Takara Bio) for cDNA synthesis.

Illumina sequence libraries were constructed from the double-stranded cDNAs. Double-stranded cDNAs were fragmented using Covaris S220 (settings: run time 55 s, peak power 175.0 W, duty factor 5.0% and 200 cycles/burst), and fragmented cDNAs were applied to KAPA Hyper Prep Kit Illumina platforms (Kapa Biosystems, Woburn, MA, United States). The quality and quantity of the Illumina libraries were evaluated using the KAPA library quantification kit (Kapa Biosystems) and applied to the Illumina MiSeq platform (Illumina, San Diego, CA, United States) according to the manufacturer’s protocol (600-cycle kit to perform 300-bp paired-end sequencing).

### Data Processing

Raw sequencing reads for dsRNA-seq were processed as described previously (Urayama et al., 2018b). rRNA reads in trimmed reads were identified by SortMeRNA (Kopylova et al., 2012) and removed. Potential genome segments were extracted from contigs, and putative RNA virus genomes were reconstructed (Urayama et al., 2020). RNA viral genes in potential genome segments and contigs were identified based on sequence similarity to known RNA viral proteins in the NCBI non-redundant (nr) database using BLASTX (Camacho et al., 2009) with an *e*-value ≤ 1 × 10^–5^. Sequences that matched a known RNA-dependent RNA polymerase (RdRp) gene by BLASTX with an *e*-value ≤ 1 × 10^–5^ were collected from RNA virus contigs and segments. In addition, a conserved domain database (CDD) search was also used. Nucleotide sequences encoding the RdRp gene were clustered at 90% identity using VSEARCH (Rognes et al., 2016).

Raw sequencing reads from ssRNA-seq were processed as described previously (Urayama et al., 2018b). Small subunit (SSU) rRNA sequences were mapped using phyloFlash (Gruber-Vodicka et al., 2019) with the option-skip_spades and -id 98. For detailed identification of major organisms, we also used EMIRGE (Miller et al., 2011) and BLASTN (Camacho et al., 2009) programs.

### Phylogenetic Analyses

Amino acid sequences of putative RdRp genes obtained in this study and their relatives in the NCBI nr database were aligned by using MUSCLE (Edgar, 2004) in MEGA6 (Tamura et al., 2013). To exclude ambiguous amino acid positions, the alignment was trimmed by trimAl (option: −gt 1) (Capella-Gutiérrez et al., 2009). Phylogenetic trees were constructed using RAxML (Stamatakis, 2014). The number of bootstrap replicates was 1000. The model of amino acid substitution was selected by Aminosan (Tanabe, 2011), as judged by the Akaike information criterion (Sugiura, 1978). MEGA6 was used to illustrate the resulting phylogeny.

### Accession Numbers

Sequences obtained in this study are available in the GenBank database repository (accession nos. DDBJ: BLWB01000001–BLWB01000058 and LC533392–LC533410) and Short Read Archive database (accession no. DDBJ: DRA009807).

## Results

### Diversity of Cellular rRNAs in Lichen

**FIGURE 2:**
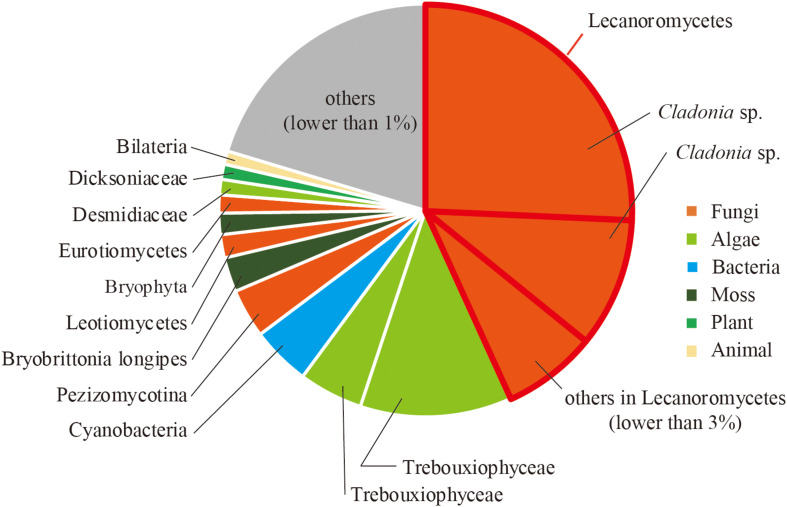
Relative abundance of sequence reads mapped on SSU rRNA sequences classified by using phyloFlash.

To reveal the composition of active microorganisms in the lichen sample, total ssRNA-seq reads were mapped on SSU rRNA sequences in the Silva database (SILVA SSU version 138) (Quast et al., 2012) using phyloFlash (Gruber-Vodicka et al., 2019), and their relative abundances were determined (Figure 2). The most abundant rRNA phylotype (43%) belonged to Lecanoromycetes (Fungi), the largest class of lichenized fungi (Miadlikowska et al., 2006), and the second abundant class was lichen-forming algae Trebouxiophyceae (Viridiplantae) (Muggia et al., 2018) that consisted of two phylotypes (17% total). These results were consistent with our morphological observation that the collected sample was a lichen. In addition to these two dominant classes, rRNA sequences from other fungi and moss were also detected (Figure 2). Classification of sequencing reads obtained by total RNA-seq is shown in Table 1.

**TABLE 1:** Classification of sequencing reads obtained by FLDS and total RNA-seq.

	dsRNA		ssRNA	
	Number of reads	Reads (%)	Number of reads	Reads (%)
Trimmed	3,955,680	100.0	1,139,888	100.0
rRNA	158,014	4.0	1,069,362	93.8
Major RNA viruses	3,340,351	84.4	2,107	0.2
Others	457,315	11.6	68,419	6.0

### Reconstruction of RNA Virus Genomes

**FIGURE 3:**
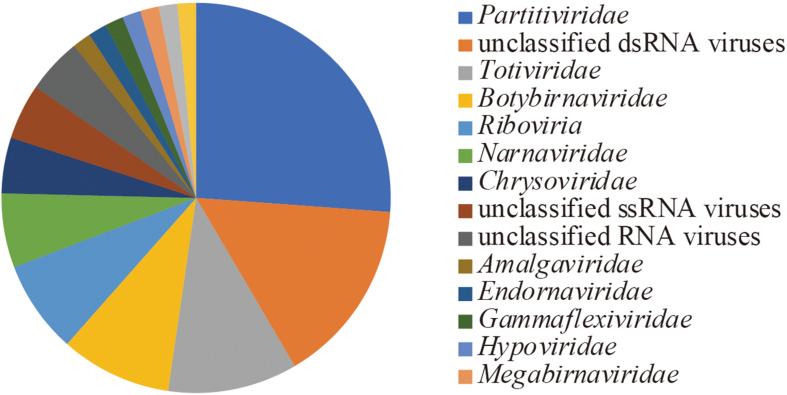
Richness of OTUs based on the taxonomic lineage of top hit sequences in BLASTX.

Following the *de novo* assembly and reconstruction of the full-length genome segments, a total of 65 RdRp-encoding operational taxonomic units (OTUs) (>1.5 kb, <90% identity between these sequences) were identified from the dsRNA-seq library (Supplementary Table 1 and described below) using BLASTX against the NCBI nr database and CDD search program. Notably, only one sequence encoding RdRp (>1.5 kb) was identified from the ssRNA-seq library, which was from Lichen partiti-like RNA virus 1 (LpaRV1) (described below). Taxonomic lineages of BLASTX top hit sequences (*e*-values ranging from 2 × 10^–30^ to 0) suggested that 17 of 65 OTUs were related to *Partitiviridae*. In addition, viruses related to seven dsRNA virus families (*Amalgaviridae*, *Botybirnaviridae, Chrysoviridae*, *Endornaviridae*, *Megabirnaviridae*, *Picobirnaviridae*, and *Totiviridae*), three ssRNA virus families (*Gammaflexviridae*, *Hypoviridae*, and *Narnaviridae*), and one unclassified RNA virus family (*Polymycoviridae*) were also identified (Figure 3).

**FIGURE 4:**
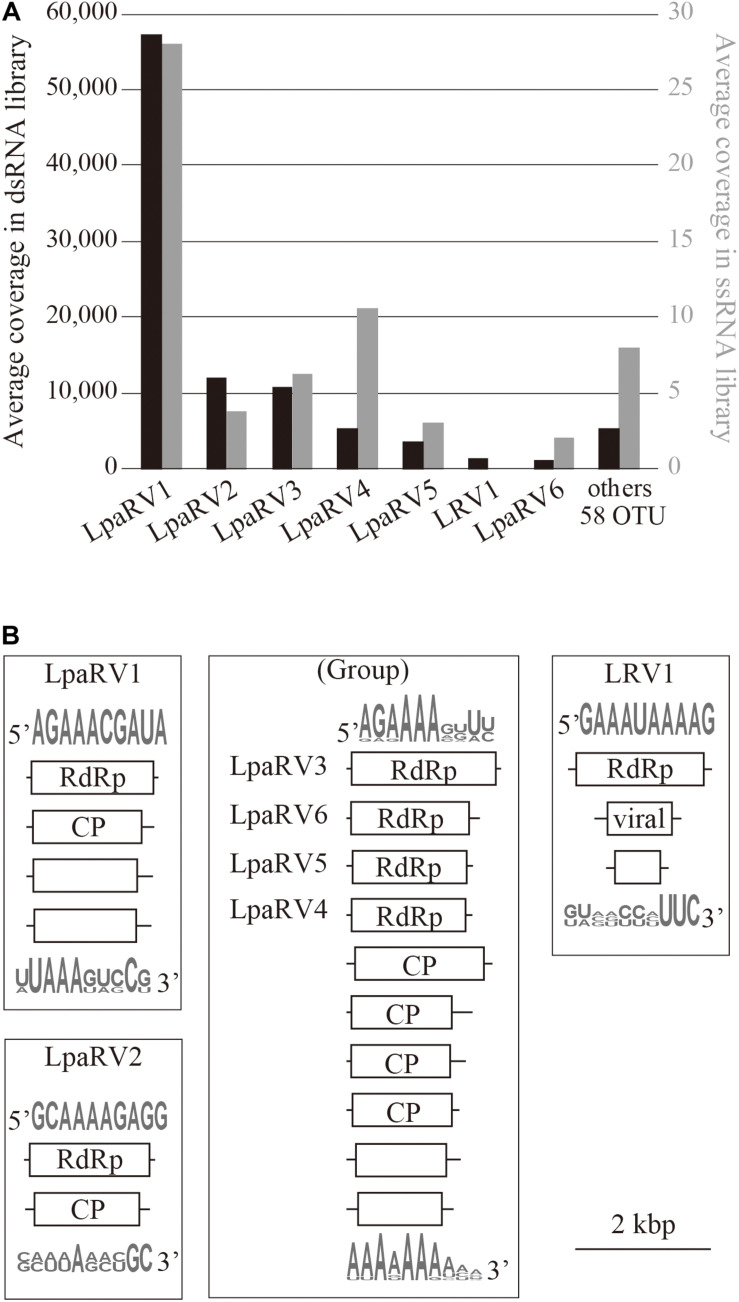
Overview of major RNA viruses identified in the lichen sample. **(A)** Average coverage of major RdRp-encoding segments in dsRNA-seq (left axis, black bar) and ssRNA-seq (right axis, gray bar) libraries. **(B)** Organization of reconstructed major RNA viral genomes. Domains identified by CDD search are shown in open reading frames (box). Sequence logos represent sequence similarities for the 5′ or 3′ terminal region of the predicted genome segments.

Among the 65 OTUs, seven full-length genome segments, whose both ends were determined to be termini based on read mapping information (Urayama et al., 2018b), showed relatively high average read coverage (>1000×) (Figure 4A) and occupied 94% average read coverage of RdRp-encoding OTUs in the dsRNA-seq library. In the ssRNA-seq library, the seven segments also occupied 87% average read coverage of RdRp-encoding OTUs (Figure 4A). Therefore, the viruses that harbored the seven dominant RdRp sequences were defined as the dominant RNA viral population in the lichen sample, and further analyses were focused on these viruses. Classification of sequencing reads obtained by FLDS is shown in Table 1.

The putative complete genome sets encoding these seven dominant RdRp sequences were reconstructed (Figure 4B and Supplementary Table 2). Terminal sequences of genome segments are shared among segments in a single virus genome in some RNA viral lineages (Hutchinson et al., 2010). Thus, we reconstructed putative genome sets based on the terminal sequences of the full-length genome segments obtained by the dsRNA-seq FLDS method. As a result, 84% of the trimmed dsRNA reads (Table 1; Supplementary Table 2) were mapped to theses major RNA viral genomes. Based on the taxonomical classification of the top hit RNA viruses in BLASTX search using entire genome segments as query sequences, these viruses were named as LpaRV1–6 and Lichen RNA virus 1 (LRV1). However, we could not distinguish the genome sets of LpaRV3–6 because the genome segments encoding their RdRps share terminal sequences. Notably, LRV1 genome sequences were not found among ssRNA reads, although LpaRV1–6 were also detected (Figure 4A).

### LpaRVs and LRV1

**FIGURE 5:**
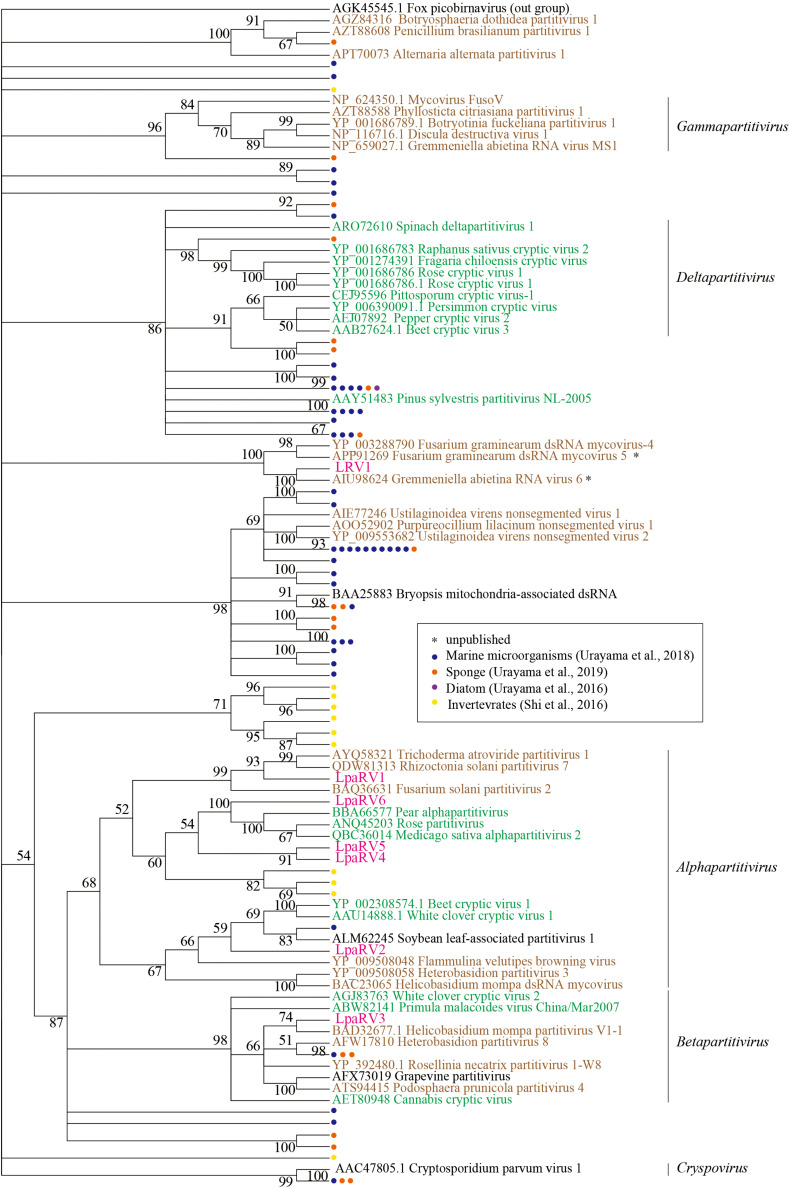
Maximum-likelihood tree of RdRp domains from representative members of the family *Partitiviridae* and related sequences (including LpaRV1–6 and LRV1) based on amino acid residues. Numbers indicate the percentage bootstrap support from 1000 RAxML bootstrap replicates. We used RAxML with the RTREV+I+G+F model. Colors of virus names indicate the classification of the host organism: green, plant; brown, fungi; black, others or unclear. Pink color represents the newly derived sequences from the lichen. All weakly supported clades (i.e., those with bootstrap support <50%) were collapsed.

LpaRV1–6 genomes consist of two to four genome segments encoding RdRp, coat protein (CP), and additional unknown proteins. The genome structures of LpaRV1–6 resemble those of partitiviruses, which are known to have a bisegmented genome encoding RdRp and CP in each segment (Nibert et al., 2014). Phylogenetic analysis of the RdRp sequences also suggested that LpaRV1–6 are members of the family *Partitiviridae* (Figure 5). To date, five genera and unidentified clades are classified into *Partitiviridae* (Nibert et al., 2014). The phylogenetic analysis of RdRp suggested that five LpaRVs (1, 2, 4, 5, and 6) are members of genus *Alphapartitivirus* and LpaRV3 belongs to genus *Betapartitivirus*. Both genera harbor viruses that infect either plants or fungi (Figure 5; Nibert et al., 2014). It is noteworthy that LpaRV1 and LRV1 located within a clade of fungus-infecting clade. Because our sample included a lot of other organisms (Figure 2), it was difficult to pinpoint the host organism, but our phylogenetic data suggested that these two viruses are infecting the fungi.

The published top hit sequence of LRV1 in the BLASTX search was Fusarium graminearum virus 4 (FgV4), which was shown to be closely related to *Partitiviridae* but not classified into the family (Yu et al., 2009). The phylogenetic analysis of partitiviruses and related viruses in this study revealed that LRV1 and FgV4 form a clade with two uncharacterized putative fungal RNA viruses, but the phylogenetic relationship among *Partitiviridae*, *Picobirnaviridae* (outgroup), and the LRV1-FgV4 cluster was not clarified. Therefore, we could not provide family-level classification of LRV1 as in the case of FgV4.

## Discussion

In this study, all dominant and putatively active RNA viruses were classified into the family *Partitiviridae* or its relative group. Among non-retro RNA viruses, only *Partitiviridae* and *Endornaviridae* harbor the genera including plant and fungal viruses. Although a single partitivirus that can infect both plant and fungal hosts has never been reported, recent studies revealed that a few viruses belonging to another RNA virus family can infect both plant and fungal hosts. For instance, cucumber mosaic virus, a positive-sense ssRNA virus belonging to the genus *Cucumovirus* in the family *Bromoviridae*, was discovered to infect both plants and fungi (Andika et al., 2017). Genome replication of a few plant viruses in *Tombusviridae* and *Bromoviridae* in a fungus (Panavas and Nagy, 2003; Panavas et al., 2005; Inaba and Nagy, 2018) and vice versa (Nerva et al., 2017) under experimental conditions was also reported. These observations suggested that the partitiviruses found in the lichen in this study might have the ability to replicate in both plants and fungi constituting symbiotic consortium of the lichen. To confirm this hypothesis, we need to conduct laboratory experiments to detect viral replication in algae cells after the infection of virus particles isolated from fungi.

It has been suggested that horizontal virus transfer among diverse hosts is one of the important driving forces of RNA virus evolution (Dolja and Koonin, 2017). In addition, Dr. Roossinck indicated that “a majority of virus families with members that infect fungi have counterparts that infect plants, and in some cases the phylogenetic analyses of these virus families indicate transmission between the plant and fungal kingdoms” (Roossinck, 2018). The strong interaction between land plants and fungi found in the symbiotic consortium of the lichen may provide an opportunity for horizontal virus transfer. In this point of view, in other fungal symbioses such as mycorrhizal fungi and their host plants, and endophytic fungi and their host plants, a similar horizontal virus transmission may occur between the symbionts. Although we could not delineate the host of each virus using our metagenomic approach, the phylogenetic information of RNA viruses associated with the lichen suggested that multiple viruses infect across higher taxonomic range between plants and fungi and impact fungi–Viridiplantae (including land plant and green algae) interaction.

Fragmented and primer ligated dsRNA sequencing provides novel insights into the RNA viromes associated with lichen. Microbial consortium of lichen would be a model system to understand virus–host coevolution. Comparison with the RNA viromes associated with the related lichens with different algae may also reveal more information about the specificity and diversity of RNA viruses and host organisms. We are also interested in the impacts of those RNA viruses on the delicate structure formation of the lichen thallus and on the symbiotic relationship between fungi and algae.

## Data Availability Statement

Sequences obtained in this study are available in the GenBank database repository (accession nos. DDBJ: BLWB01000001–BLWB01000058, LC533392–LC533410) and Short Read Archive database (accession no. DDBJ: DRA009807).

## Author Contributions

SU, TN, ND, and YM designed the research. FK and MH performed the research. ND and YM collected samples. YT, YC, and SU analyzed the data. All the authors listed wrote the manuscript. All authors contributed to the article and approved the submitted version.

## Conflict of Interest

This research is a joint project funded by Nippon Gene Co., Ltd. In this research, we used ISOVIRUS, a product sold by Nippon Gene Co., Ltd. YM and ND are employed by Nippon Gene Co., Ltd. The remaining authors declare that the research was conducted in the absence of any commercial or financial relationships that could be construed as a potential conflict of interest.

## Supplementary Material

Supplementary Table 1 A list of RdRp-encoding OTUs except for major RNA viruses.

Supplementary Table 2 Putative complete genomes of RNA viruses obtained from a lichen.
